# Week Long Topography Study of Young Adults Using Electronic Cigarettes in Their Natural Environment

**DOI:** 10.1371/journal.pone.0164038

**Published:** 2016-10-13

**Authors:** R. J. Robinson, E. C. Hensel, K. A. Roundtree, A. G. Difrancesco, J. M. Nonnemaker, Y. O. Lee

**Affiliations:** 1 Department of Mechanical Engineering, Rochester Institute of Technology, Rochester, New York, United States of America; 2 Research Triangle International, Research Triangle Park, North Carolina, United States of America; Legacy, Schroeder Institute for Tobacco Research and Policy Studies, UNITED STATES

## Abstract

Results of an observational, descriptive study quantifying topography characteristics of twenty first generation electronic nicotine delivery system users in their natural environment for a one week observation period are presented. The study quantifies inter-participant variation in puffing topography between users and the intra-participant variation for each user observed during one week of use in their natural environment. Puff topography characteristics presented for each user include mean puff duration, flow rate and volume for each participant, along with descriptive statistics of each quantity. Exposure characteristics including the number of vaping sessions, total number of puffs and cumulative volume of aerosol generated from ENDS use (e-liquid aerosol) are reported for each participant for a one week exposure period and an effective daily average exposure. Significant inter-participant and intra-participant variation in puff topography was observed. The observed range of natural use environment characteristics is used to propose a set of topography protocols for use as command inputs to drive machine-puffed electronic nicotine delivery systems in a controlled laboratory environment.

## Introduction

### Background and Rationale

Electronic Nicotine Delivery Systems (ENDS) sales, marketing and use have proliferated in the United States and globally since their introduction into the marketplace. ENDS are reported as the most commonly used tobacco product among youth in the U.S. [1], suggesting that these products will become increasingly prevalent. This is of concern because scientists have not yet determined the potential harms or benefits ENDS may have on both individual-level and population-level health. Improved understanding of these potential harms or benefits is critically necessary to inform the public health response to these products by regulatory and policy-making agencies. As of May 2016, the Food and Drug Administration (FDA) is authorized to regulate ENDS products and is charged with establishing testing and product standards [2] [3] [4]. These standards will be informed in-part by scientific findings on user behavior and puffing topography. Data on use behavior and puffing topography are essential for accurately assessing exposures to ENDS emissions constituents but currently few studies exist. A key barrier to this assessment is the limited measurement of ENDS topography and lack of established testing protocols. Accurate user driven measurement of ENDS topography is also crucial for understanding the health effects of these products. For example, conventional cigarette smokers were found to take larger, longer and more frequent puffs when smoking “low-yield” cigarettes thus resulting in similar toxicant delivery as cigarettes that were not “low-yield”[5, 6]. Indeed, regulations from the 1970’s, put in place to standardize testing of combustible tobacco cigarettes, did not include realistic topography protocols and were eventually rescinded [7]. This study aims to support regulatory science by contributing data that describes realistic topography for ENDS users. Assessing the topography associated with ENDS use is critically important for developing studies that can provide meaningful risk assessment of ENDS use such as *in vitro* and *in vivo* studies that assess ENDS emissions exposure.

While conventional cigarette smoking behavior has been studied extensively [8–18], fewer studies are available on direct measurement of ENDS puffing topography. Results from ENDS topography studies suggest that conventional cigarette and ENDS topographies are different [19, 20]. The emerging literature on ENDS topography also consists of highly varied studies that feature different limitations, such as use environment, measurement devices and monitoring duration, making comparisons across studies challenging. Of the studies listed in Table 1, seven were conducted in the laboratory environment [21], [22], [20], [20], [23], [24], [25]and only one [26] was conducted in the natural environment. Lab environment studies are limited in their ability to monitor natural behavior and therefore the resulting puff topography characteristics and any exposure data derived from them may not accurately represent actual patterns of users [27]. The monitoring devices used to record puffing topography also vary across studies and result in various limitations. The CReSS device (Plowshare, Inc.) utilized in two of four studies has several reported limitations. In particular, device failure causing loss of data was reported in one study [28] and inaccurate puff counts, and maximum recordable puffs limit of 43 puffs was reported in another study [22]. The EVIC, an ENDS manufactured by Joyetech that records puff duration, was used in one study [23] to measure puff duration, but without the knowledge of puff flow rate or puff volume, puff topography cannot be fully characterized. Ideally results from topography studies should accurately represent puffing behaviors of users in their natural environment, so it is preferential to capture user’s behavior in the natural environment, but until recently lack of adequate technology limited the studies to the lab environment. Robinson *et al*. [26] recently demonstrated the gathering of comprehensive ENDS puffing topography and usage data outside the laboratory environment and provided preliminary data on inter- and intra-participant variability in puffing characteristics for 21 users over a 24 hour period. The current study applies the techniques described in Robinson *et al*. to a week-long monitoring study on ENDS users’ vaping in their natural environment. Both the natural environment and the extended monitoring period provides enhanced confidence that the topographies collected in the present study will represent realistic vaping patterns.

**Table 1:** Previous studies involving ENDS topography measurements.

Study	Number of Participants	Testing Conditions	Mean Puff Duration / inter-puff gap *(sec)*	Mean Flow Rate *(mL/sec)*	Mean Puff Volume *(mL)*	Method
Goniewicz *et al*. 2013 (*a*,*b)*	N = 10	Lab, fixed number (15± 6) of puffs per session, fixed time between sessions	1.8 ± 0.9 (STD) / 10 ± 13 (STD)	10 ± 13 (STD)	70 ± 68 (STD)	CReSS
Spindle *et al*. 2015 *(b)*	N = 13	Lab, fixed number of puffs and fixed inter-puff	4.16 ± 1.06 (*NR*) / *NR*	24.17 ± 10.66 (*NR*)	101.37 ± 50.01 (*NR*)	In-house device
Norton *et al*. 2014 *(a)*	N = 18*	Lab, *ad lib*	3.0 ± 0.8 (SE) / 29.6 ± 11.7 (SE)	52.0 ± 4.7 (SE)	118.2 ± 13.3 (SE)	CReSS
Behar *et al*. 2015 *(a)*	N = 20	Lab, *ad lib* puffing for 10 min	2.65 ± 0.98(STD) /17.9 ± 8.4 (STD)	20 ± 6 (STD)	51 ± 21 (STD)	CReSS
Farsalinos *et al*. 2015 *(b)*	N = 24 N = 23*	Lab: fixed number of puffs in 5 minutes; then *ad lib* puffing for 60 min. Total duration 65 minutes.	*3*.*5 ± 0*.*2 (NR) 2*.*3 ± 0*.*2 (NR) / NR*	*NR*	*NR*	EVIC
Lee *et al*. 2015 *(a)*	N = 20*	Lab, *ad lib* for 1 session with no fixed time	2.9 ± 0.2 (SEM) / 18.8 ± 3.3 (SEM)	24.8 ± 1.9 (SEM)	63.3 ± 5.2 (SEM)	CReSS
*Lopez et al*. *2016 (b)*	N = 16*	Lab, fixed number of puffs per session (10), fixed time between sessions (30 sec)	2.85 ± 1.49 (STD) / *NR*	27.1 ± 13.1 (STD)	70 ± 28.8 (STD)	*NR*
Robinson *et al*. 2015*(a)*	N = 22	Natural environment	3.5 ± 0.4 (SEM) / NR	*37* ± 3.5 (SEM)	133 ± 20 (SEM)	wPUM

Note:

* indicates Naïve ENDS users. STD = standard deviation, SE = standard error, SEM = standard error Mean, NR = value or error type not reported. Study Notes: (a) first generation / ‘cigalike’ electronic cigarettes (b) second generation / tank style electronic cigarettes.

### Objectives

The current study aims to enhance current understanding of ENDS puffing topography and use behavior as described by puff duration, puff volume and puff flow rate, and cumulative exposure to aerosol generated from ENDS use (e-liquid aerosol). The current study aims to improve understanding of both intra-participant variability (variation in behavior for a given participant) and inter-participant variability (variation in behavior among participants in a given cohort). The ultimate goal of this research is to inform regulation of ENDS by (1) providing more accurate puffing topographies for use in machine generated emission studies, (2) providing realistic cumulative exposure data for use in *in vitro* and *in vivo* exposure studies, and (3) providing insight into the natural variation in use behavior for individual participants and participant cohorts for use in designing human monitoring and product switching studies.

## Methods

### Study Design

This is an observational descriptive study of experienced adult ENDS users. The study asked participants to use their own ENDS as normal with a wireless hand-held monitoring device for a one week period. Participants were recruited at the Rochester Institute of Technology (RIT), Henrietta campus and data was collected from twenty participants between April 7, 2015 and May 21, 2015.

### Cohort Recruitment and Protocol

The study protocol, including participant recruitment, informed consent, waiver to participate, survey instrument, participant testing schedule, advertising, exclusion criteria and the study purpose were reviewed and approved by the Rochester Institute of Technology (RIT) Human Subjects Research Office Institutional Review Board (IRB) and the RTI International IRB. The participants participation consisted of: written informed consent, surveys, wireless Personal Use Monitor (wPUM) training, wPUM usage, and device return. Participants were recruited to the study through the use of posters placed around campus. The RIT campus population consists of approximately 15,400 undergraduate students, 3,200 graduate students, and 3,800 faculty and staff. The student population is largely full-time and residential. Home to the National Technical Institute for the Deaf, the student population includes approximately 1,200 deaf and hard-of-hearing students. The posters advertised a research study regarding electronic cigarettes and stated that participants would get a monetary incentive for participating. The poster also provided an email for the interested individuals to request more information. Everyone who sent an email to the address received the same response which provided a detailed summary of the study, and highlighted what was required of the participant if they chose to participate. They were also told that to participate in the study they must currently be an electronic cigarette user, have had experience using either disposable or rechargeable electronic cigarettes, and they would be paid $200 if they participated and correctly fulfilled their requirements for the study. Finally, they were asked to reply via email if they were still interested in participating in the study.

Each participant was scheduled a time to participate in the study based on their availability. The participants were surveyed on smoking history and behavior. Prospective participants were screened for current use of ‘first generation’ or cigalike devices that were either disposable or rechargeable and at least three months of prior ENDS use experience. Next, each participant attended an individual training session on how to use the wireless Personal Use Monitor (wPUM, described below), conducted in the Respiratory Technology Lab (RTL) at RIT. At the conclusion of the training session, each participant was provided with a packet of instructions for how to correctly use the monitor in case they forgot something covered during the training. The participants were then provided with the wireless personal use monitor. The participants then left the RTL with the instructions to use the wPUM for each puffing event for *1 week*. The participants were asked to use their preferred ‘first generation’ or ‘cigalike device’ (disposable or rechargeable ENDS). The participants were asked to return the wPUM to the RTL after the *1 week* trial, at a pre-determined time, that was convenient for them. The participants were also required to come to the RTL laboratory three days after their initial start date in order for the researchers to affirm that the wPUM was working properly and perform routine device maintenance as required.

### Wireless Personal Use Monitor

**Fig 1:**
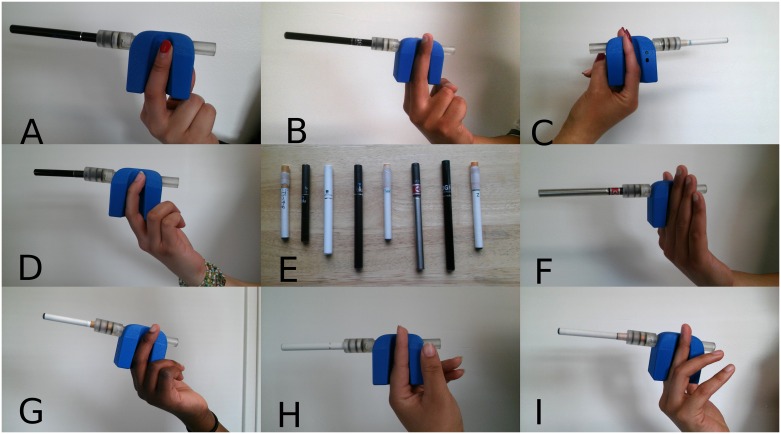
Wireless Personal Use Monitor (wPUM) with various commercially available ENDS. The wPUM is designed with an aperture that can accommodate a variety of ENDS and conventional cigarettes. The wPUM records digitized signals at the rate of 40 samples/sec to the memory of the device.

Puffing topography was measured with a wireless personal use monitor (wPUM) designed, built and tested at RIT and described in a previous paper [26]. The wPUM (Fig 1) is a portable unit, and a cigarette aperture that can accommodate multiple brands of ENDSs and conventional cigarettes. In the current study, the wPUM was used for ‘1^st^ generation’ or ‘cigalike’ devices. However, the wPUM is adaptable and has been applied to a wide range of devices. A future study will report topography results from other categories of ENDS devices. The wPUM utilizes proven orifice plate technology to measure puff flow rate as described previously [26]. A standardized calibration protocol is used to convert the digitized voltage signal to flow rate as measured by an Alicat flow meter. The standardized calibration protocol includes *36* puffs of *5 sec* duration, separated by *5 sec* and ranging from *10 mL/s* to 6*0 mL/s*, such that each flow rate is repeated six times during the calibration sequence. The calibration coefficient is determined by a customized calibration program employing linear regression.

The wPUM begins recording when the device is turned on and continues recording until the device is turned off, and results in one data file. Multiple data files can be stored on the wPUM so that a user can be monitored in their natural environment for multiple days at a time without intervention of a research administrator. At the conclusion of the observation period, puff monitoring data is exported from the wPUM and analyzed with the RIT Topography Analysis Program (TAP). The process for analyzing data is described in the next section.

### Topography Analysis Program

Monitoring data is analyzed with the Topography Analysis Program (TAP) developed in-house by an investigator from the Respiratory Technologies Laboratory at Rochester Institute of Technology. The TAP identifies discrete puffs, calculates topography characteristics and tabulates and plots descriptive statistics. A preliminary analysis is completed in which an analyst inspects each data file generated by the wPUM device for data integrity. During this process, the analyst makes note of any data files that may have included spurious data lines (such as an incomplete data line at the end of the file as the wPUM powered down), files lasting for many hours (suggesting the device may have been turned on inadvertently, and therefore should not be counted as an actual session, or not turned off after a session was complete and therefore may contain multiple puffing sessions in one file), and those files containing unusual use characteristics which deserve further investigation (such as unusually high levels of noise and drift in the baseline voltage). All data files are preserved in their original form. The analyst may elect to exclude individual data files based on any of the exemplar reasons listed; the reason for excluding any single data file is documented and justified in the analysis protocol employed for each test participant. After preliminary analysis is complete, the TAP is used to perform a sequence of signal processing steps, using instantaneous flow rate and cumulative volume to detect and quantify puff topography characteristics, as described previously [26], [29].

The TAP plots out the raw signal measured for every session (puff flow rate versus time) overlaid with the derived puffing topography based on the TAP analysis [26]. For each puff discretely identified by the TAP, the program calculates puff duration, puff volume, puff flow rate (computed as the mean flow rate yielding the observed cumulative volume over the observed duration of the puff), and puff interval and tabulates these along with the date and time stamp for each puff for each participant. The analyst may review and confirm each puff, and adjust TAP parameters to ensure that all data sets are analyzed consistently. The TAP tabulates average topography characteristics and cumulative weekly exposure (number of puffs and total volume) by session and by week. The TAP also computes the standard deviation, standard error of the mean, *95%* Confidence Interval on the mean and variance and creates interval plots, box plots and frequency distributions. Optional statistical tests may be computed with the TAP, comparing mean puff duration, puff flow rate, and puff volume of any participant with those of any other participant in the study cohort. The TAP includes a parameter enabling the analyst to set a “maximum duration” cut-off on any individual puff so that descriptive statistics can be presented on a subset of topographies determined by the analysist to be indicative of reasonable participant use behavior. The analysis code reports details of all data and files processed and excluded including puffs that are eliminated under the “maximum duration” cut-off conditions. Neither the wPUM nor the TAP impose an inherent limit on the maximum duration of puffs. The ability to articulate a maximum puff duration in the analysis permits the analyst to conduct sensitivity analyses of topography parameters in relation to the maximum duration permitted. Results presented herein are subject to a *20 [s]* maximum puff duration. A preliminary sensitivity analysis confirmed that reducing the maximum puff duration below 20 [s] could have an observable influence on the resulting mean puff duration and volume. Conversely, puffs above 20 [s] were clearly outliers and had minimal impact.

## Results

### Study Cohort

**Fig 2:**
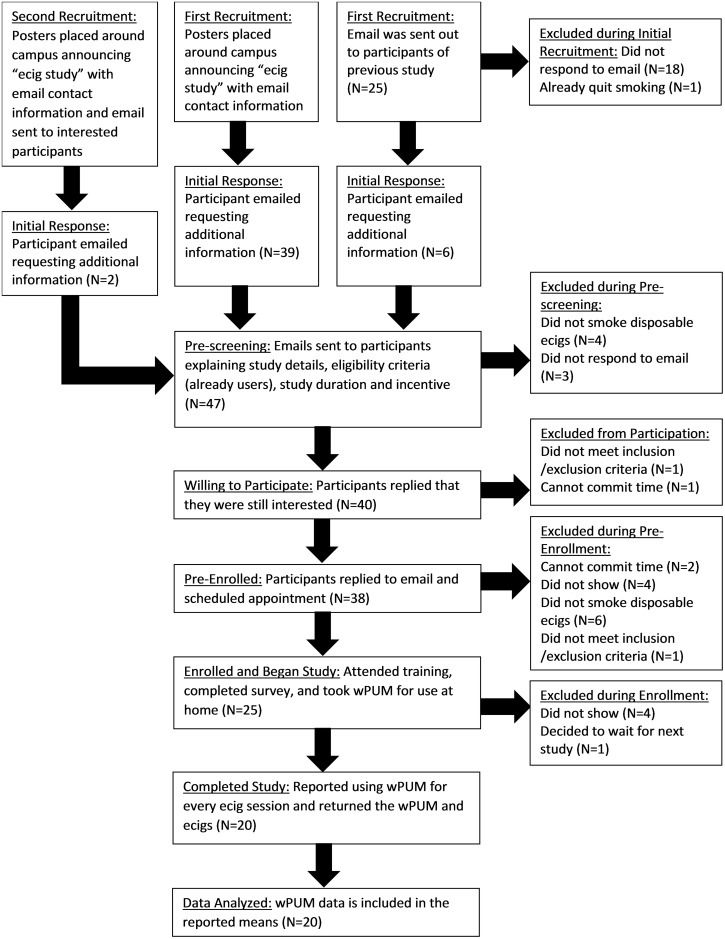
Cohort study flow chart. Forty-seven individuals responded to the initial recruitment. Data for all twenty participating participants are reported in this study. No participants who participated are excluded from the data presentation.

A total of 47 respondents requested information about study participation. The progress of these 47 individuals through the screening process and into the study is shown in Fig 2. Of the initial 47 respondents, 4 were ineligible during pre-screening because they were not ENDS users, and 3 did not confirm continued interest in participation. From the remaining 40, 38 scheduled a pre-enrollment date. During the pre-enrollment period, 2 participants were excluded as unable to meet the time commitment, 6 were excluded for not using disposable or rechargeable ENDSENDS products, 4 did not respond to the pre-enrollment email and 1 did not meet the inclusion criteria. Twenty five participants were schedule for appointments to be enrolled in the study; 4 individuals did not appear for their enrollment appointment and 1 individual cancelled his enrollment appointment because he decided to wait for the next study.

Of the 20 participants completing final enrollment, 19 were male and 1 was female, and they ranged in age from 18 to 22 years. Two participants had never smoked traditional cigarettes before, and 10 participants had smoked traditional cigarettes for at least one year. Of the 18 participants who had smoked traditional cigarettes before, 9 were former smokers, 1 was current smoker on a daily basis, and the remaining 8 reported less-than-daily use of combustible cigarettes. Each participant indicated the brand of disposable or rechargeable ENDS they would be using for the duration of the week long natural use environment observation period and prior ENDS brand(s) used. Information collected from each participant during the enrollment interview is presented in Table 2. All 20 participants completed the 1 week trial, returning for their 3-day wPUM maintenance check and returning the wPUM to the lab at their scheduled appointment time.

**Table 2:** Participant information collected during the enrollment interview.

Participant	Age *(yrs)*	Participant’s preferred brand (and type) used during the study *(-)*	Nicotine Strength marked on packaging.	Note
1	19	Blu (GEN1)	17–24 mg	
2	18	Logic (GEN1)	2.4%	
3	18	Zoom (GEN1)	3.5	2
4	22	V2 (GEN1)	0.6%	
5	20	Logic (GEN1)	1.8%	1, 3
6	20	Logic (GEN1)	1.8%	1
7	18	Markten (GEN1)	2.5%	1, 3
8	21	Vuse (GEN1)	4.8%	3
9	19	Blu (GEN1)	16 mg	
10	20	Njoy (GEN1)	NR	
11	19	Logic (GEN1)	2.4%	2
12	22	Vuse (GEN1)	4.8	
13	20	Blu (GEN1)	16 mg	3
14	19	Logic (GEN1)	3 mg	1
15	22	Blu (GEN1)	17–24 mg	
16	18	Blu (GEN1)	NR	1
17	19	Zoom (GEN1)	4.40%	2
18	19	Criss Cross (GEN1)	High	1
19	19	Criss Cross (GEN1)	High	1
20	21	Vuse (GEN1)	24mg (2.4%)–reported by participant	1

Participant-reported information regarding demographics and ENDS use patterns and preferences. Notes: (1) participant switched back for this study, (2) participant reported using multiple ENDS brands regularly, (3) rechargable brand of ENDS. NR = value not reported on package.

### Preliminary Data Integrity Analysis

The wPUMs were collected from each participant at the conclusion of the week-long observation period. Data files for the *7 day* observation period were downloaded from the wPUM for preliminary analysis. During the preliminary analysis process data files were screened to insure data integrity. Only those files deemed to include quality puffing data were read by the TAP and included in the topography statistics. Table 3 shows the results of the preliminary analysis conducted on each participants’ data set. Included in Table 3 are the number of data files download from the wPUM and inspected for quality, and the number of files passing the quality check and analyzed by the TAP. In total, 1022 downloaded data files were inspected for quality, and 90% of these files or 922 total files were deemed to contain quality data and therefore analyzed by the TAP. There were 9 Participants who had 100% of their data files pass the quality phase of the preliminary analysis. The remaining participants had files excluded for quality control reasons described below.

**Table 3:** Preliminary Data Integrity Analysis for participants 1 through 20.

Participant	Total files from wPUM *(-)*	Files included after quality review *(-)*	Percent of files included *(%)*
1	34	34	100%
2	111	91	82%
3	34	34	100%
4	10	10	100%
5	265	261	98%
6	79	65	82%
7	33	17	52%
8	74	69	93%
9	24	24	100%
10	18	18	100%
11	55	50	91%
12	53	35	66%
13	10	9	90%
14	81	68	84%
15	34	34	100%
16	21	19	90%
17	23	23	100%
18	34	32	94%
19	12	12	100%
20	17	17	100%
Cohort Totals	1022	922	90%

Summary of data collected with the wireless Personal Use Monitor (wPUM) for 20 participants across multiple puffing sessions conducted during 7 days of ENDS use in the natural use enviroment. The second column indicates the number of topography data files downloaded from the wPUM and inspected for quality. The third column indicates the number of files passing the quality check and analyzed by the Topography Analysis Program (TAP). The fourth column indictes the percentage of files passing the quality check.

When Participant 7 returned the wPUM to the lab, the technician observed that a hose connected to one end of the orifice plate inside the wPUM had become disconnected. This was evident in 16 files from the later portion of the week, so those files were excluded from the analysis for Participant 7. Participant 12 presented 18 files exhibiting significant drift in the baseline voltage recorded with the wPUM during the first half of the week. During a mid-week consultation with Participant 12, a calibration was conducted on the wPUM, and a droplet of e-liquid was observed to be entrained in the wPUM tubing. The wPUM was cleaned, calibrated, and returned to the user. Participant 12 files for the remainder of the week were observed to contain expected characteristics typical of puffing. Participant 6 presented 14 files having apparently inverted puff profiles, dissimilar to topography data previously observed with the wPUM in a controlled laboratory setting, so those files were excluded from the analysis for Participant 6, pending further investigation. Participant 2 presented 20 files observed to have a combination of the baseline drift evident in the files of Participant 12 and inverted profiles evident in the files of Participant 6. Participant 14 presented 13 files having a similar combination of baseline drift and inverted profiles. Participants 5, 8, 11, 13, 16 and 18 presented a small number of files exhibiting baseline drift and occasional inverted profiles, resulting in 2% to 10% of those participant’s files being excluded from the statistical analysis presented herein. After the observation period concluded, an investigation was conducted to reproduce the source of voltage bias and inverted profiles. Preliminary results of that investigation, supported by the ability to reproduce such characteristics in a controlled setting, suggest that the voltage bias and inverted puff profile may be the result of ENDS liquid entrained in or condensed within the wPUM. Work is currently under way to compensate for condensate in an effort to recover partially useful information from the excluded files.

The TAP program was run on the 922 files that passed the initial data integrity check. The TAP identified 134 of the 922 files as containing zero puffs. Close inspection of these 134 data files indicated that the wPUM was turned on but no actual vaping session took place. These “zero-puff” files were not counted in the participants’ vaping session totals. Note that as a result, the total vaping sessions per participant is less than the total number of files analyzed by the TAP. Also note, preliminary analysis revealed that in some cases a participant turned on the wPUM, took some puffs during a short time interval, left the device on for an extended period of time, and then took a few more puffs during a short time interval before turning the wPUM off. Such an event is reported as a single vaping session in this study. In summary, data included in the topography characteristics and cumulative exposure assessment were analyzed and deemed to be measures of actual vaping activity and analyzed accordingly.

### Vaping Session Profiles

**Fig 3:**
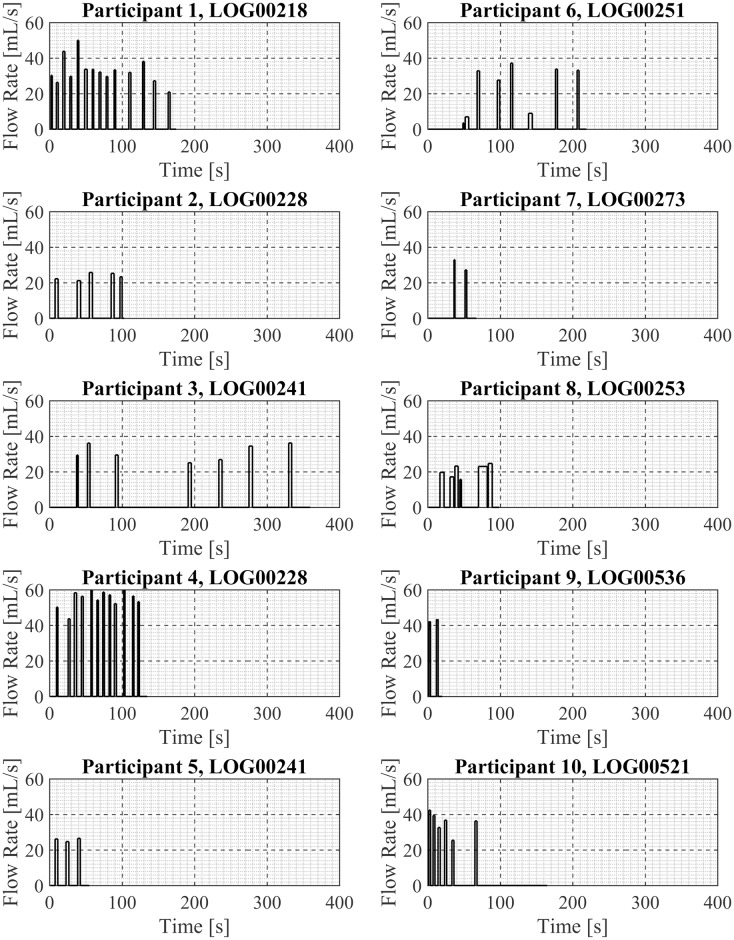
Exemplar puffing profiles for participants 1 through 10. Each panel presents analysis results of a single exemplar puffing session selected from among *7 days* of natural use enviroment ENDS puffing sessions by a single participant. The plots in each panel illustrates the mean puff flow rate, puff start time and puff end time as a function of time since the beginning of the puffing session.

**Fig 4:**
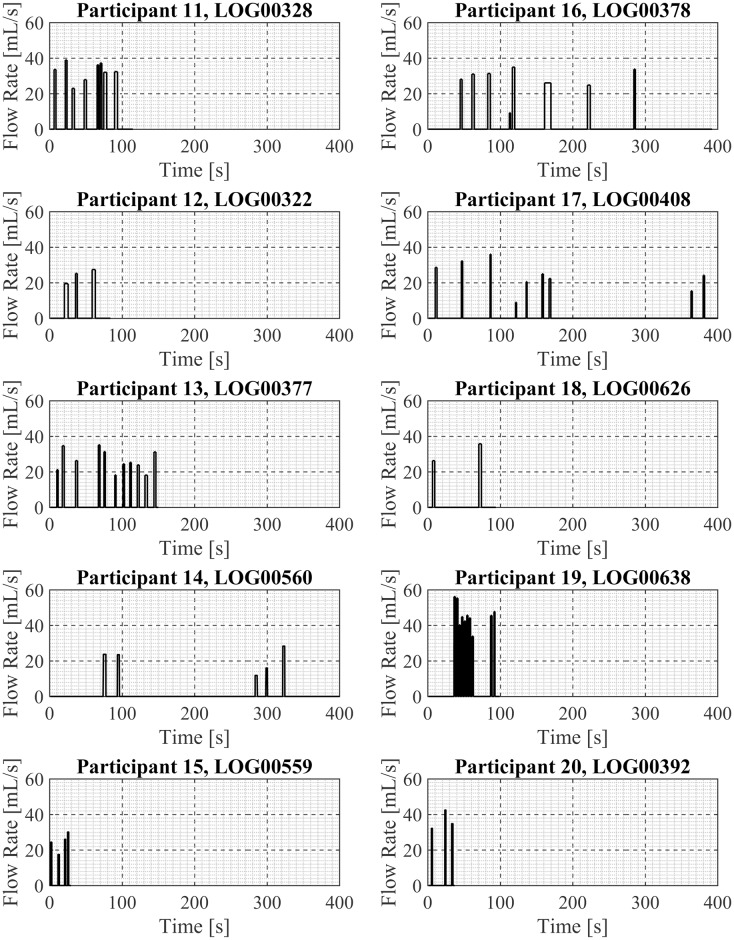
Exemplar puffing profiles for participants 11 through 20. Each panel presents analysis results of a single exemplar puffing session selected from among *7 days* of natural use enviroment ENDS puffing sessions by a single participant. The plots in each panel illustrates the mean puff flow rate, puff start time and puff end time as a function of time since the beginning of the puffing session.

**Fig 5:**
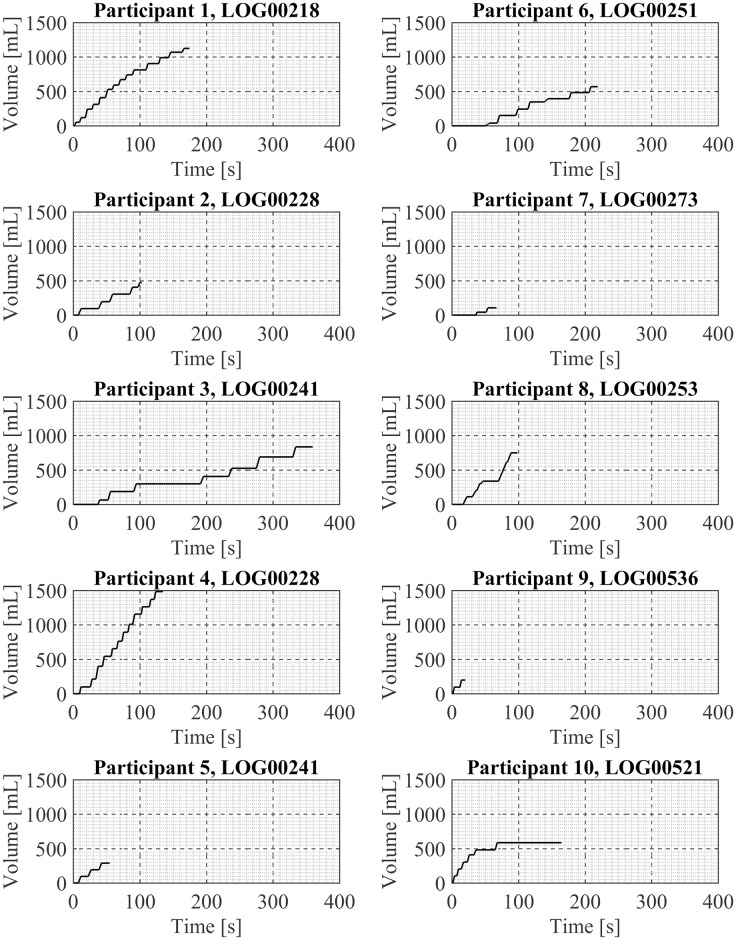
Cumulative volume inhaled during exemplar puffing sessions shown in Fig 3 for participants 1 through 10. Each panel presents analysis results of a single exemplar puffing session selected from among *7 days* of natural use enviroment ENDS puffing sessions by a single participant. The plots in each panel illustrates the cumulative volume inhaled by the participant with each subsequent puff during the session.

**Fig 6:**
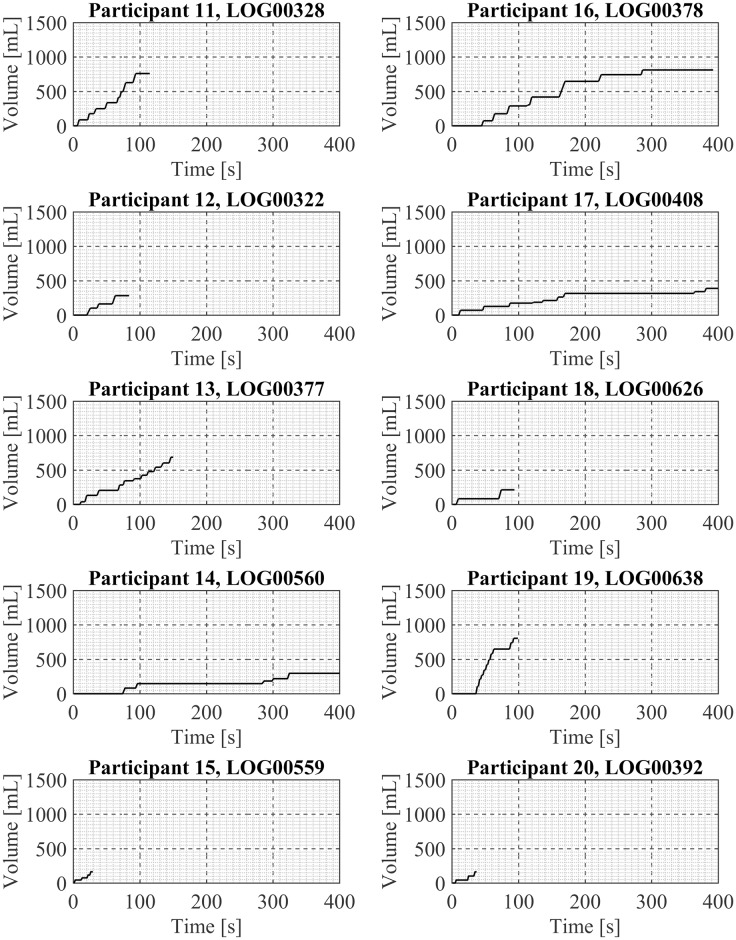
Cumulative volume inhaled during exemplar puffing sessions shown in Fig 4 for participants 11 through 20. Each panel presents analysis results of a single exemplar puffing session selected from among *7 days* of natural use enviroment ENDS puffing sessions by a single participant. The plots in each panel illustrates the cumulative volume inhaled by the participant with each subsequent puff during the session.

One exemplar puffing session is presented here for each of the 20 participant in Figs 3 and 4, in sets of 10 participants per figure. Shown are the mean puff flow rate and the puff duration of each discrete puff taken by the participant during the exemplar puffing session. Participant. Examination of these exemplar puffing sessions illustrates the various vaping patterns exhibited by these 20 first generation ENDS users in their natural environment. For example, there are sessions containing three or fewer puffs (Participants 5, 7, 9, 18, 20) and sessions containing over a dozen puffs (Participants 1, 4, 19). There are relatively short-duration sessions of less than 20 seconds (Participant 9) and relatively long-duration sessions (Participants 14, 17) of more than 400 seconds duration. Some sessions show regular intervals between puffs (Participant 20) while other sessions show irregular intervals between puffs (17). Some sessions (Participant 14) have “sets” of puffing activity where puffs were taken in a short time frame, approximately 15 sec apart, followed by a break of 3minutes and then another set of puffing activity with several puffs taken 15 to 20 seconds apart. The cumulative session volume (the integral of the flow rate curve) associated with each participant’s exemplar puffing session (previously shown in Figs 3 and 4) is illustrated in Figs 5 and 6. Examination of Figs 5 and 6 illustrates the wide range of exemplar volume exposures across the 20 participants. For example, the cumulative volumes in the exemplar sessions ranged from less than 200 ml (Participants 7, 9, 15, 18, and 20) to over 1200 ml (Participants 1 and 4). Some sessions had small inhaled volumes over short durations (Participants 7, 9, 5, 12, 15 and 20) while other sessions had small inhaled volumes over much longer durations (Participants 14 and 17). These exemplar sessions illustrate the type of vaping patterns that were captured by the wPUM for ENDS users who were vaping their preferred first generation (disposable or rechargeable) product in the natural environment.

### Intra-participant Variability

**Fig 7:**
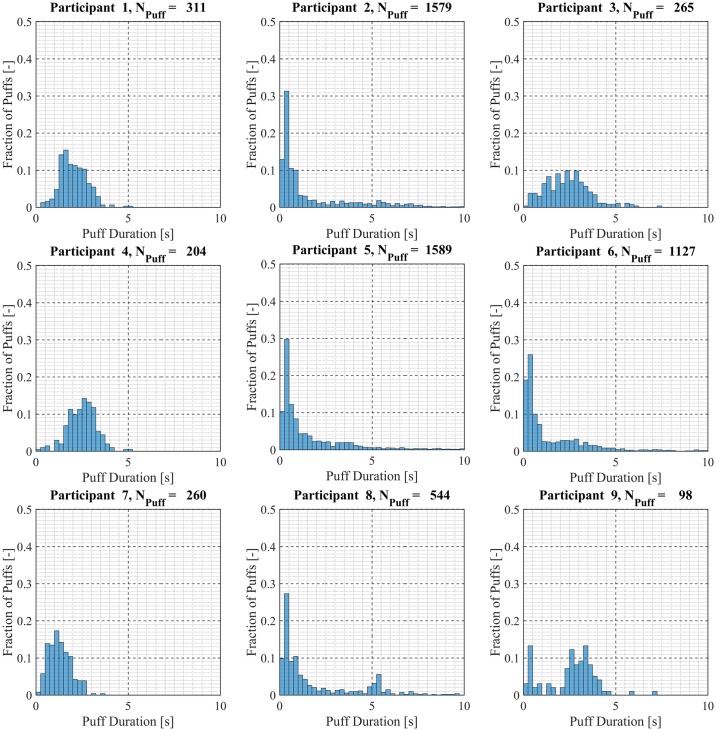
Frequency distributions of puff duration variation for participants 1–9. Each frequency distribution panel presents the variation of puff duration of a single participant across multiple puffing sessions conducted during *7 days* of ENDS use in the natural use enviroment.

**Fig 8:**
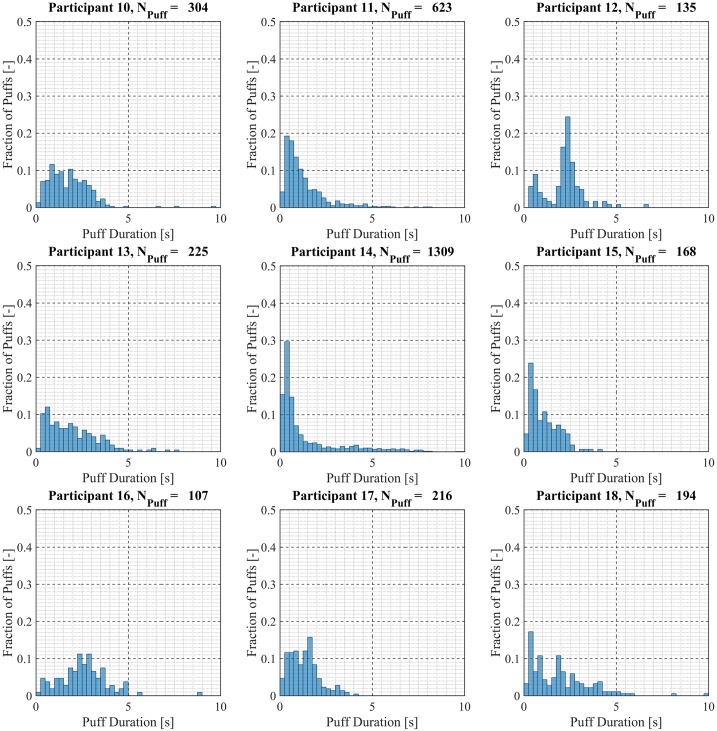
Frequency distributions of puff duration variation for participants 10–18. Each frequency distribution panel presents the variation of puff duration of a single participant across multiple puffing sessions conducted during *7 days* of ENDS use in the natural use enviroment.

**Fig 9:**
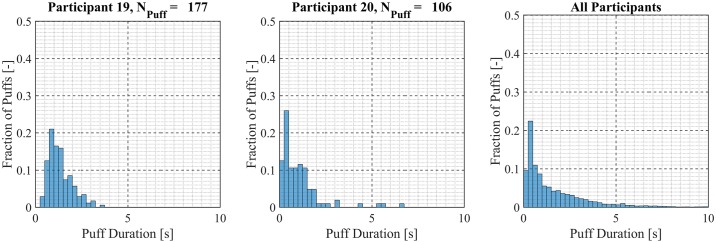
Frequency distributions of puff duration variation for participants 19–20. Each frequency distribution panel presents the variation of puff duration of a single participant across multiple puffing sessions conducted during *7 days* of ENDS use in the natural use enviroment. The right column panel presents puff duration variation for the entire cohort of 20 participants.

**Fig 10:**
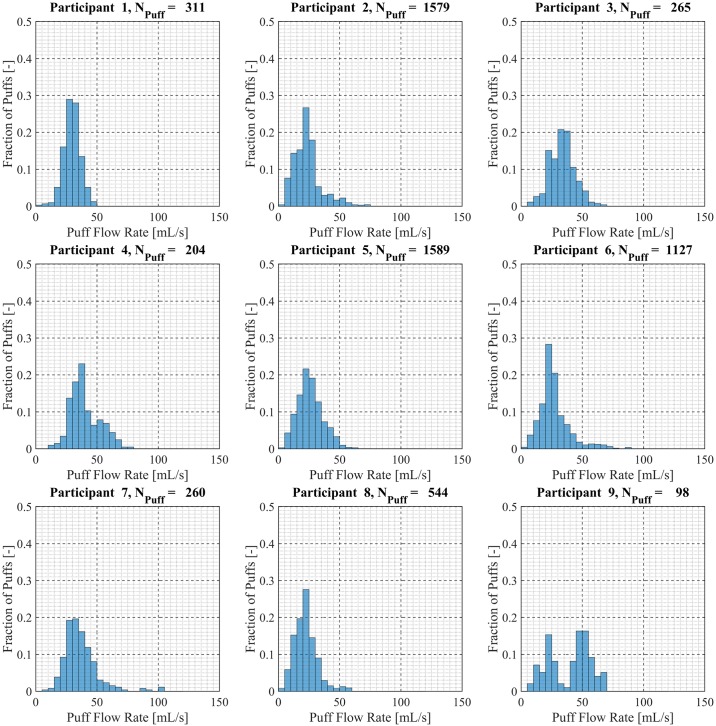
Frequency distributions of puff flow rate variation for participants 1–9. Each frequency distribution panel presents the variation of puff flow rate of a single participant across multiple puffing sessions conducted during *7 days* of ENDS use in the natural use enviroment.

**Fig 11:**
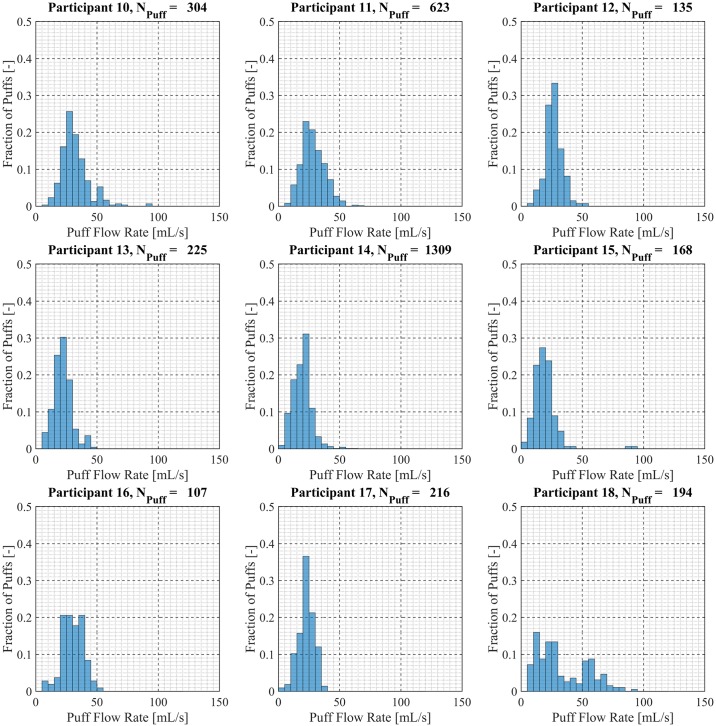
Frequency distributions of puff flow rate variation for participants 10–18. Each frequency distribution panel presents the variation of puff flow rate of a single participant across multiple puffing sessions conducted during *7 days* of ENDS use in the natural use enviroment.

**Fig 12:**
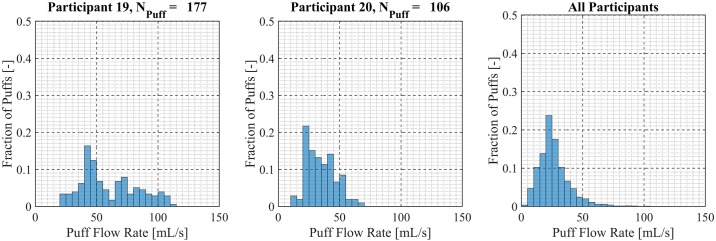
Frequency distributions of puff flow rate variation for participants 19–20. Each frequency distribution panel presents the variation of puff flow rate of a single participant across multiple puffing sessions conducted during *7 days* of ENDS use in the natural use enviroment. The right column panel presents puff flow rate variation for the entire cohort of 20 participants.

**Fig 13:**
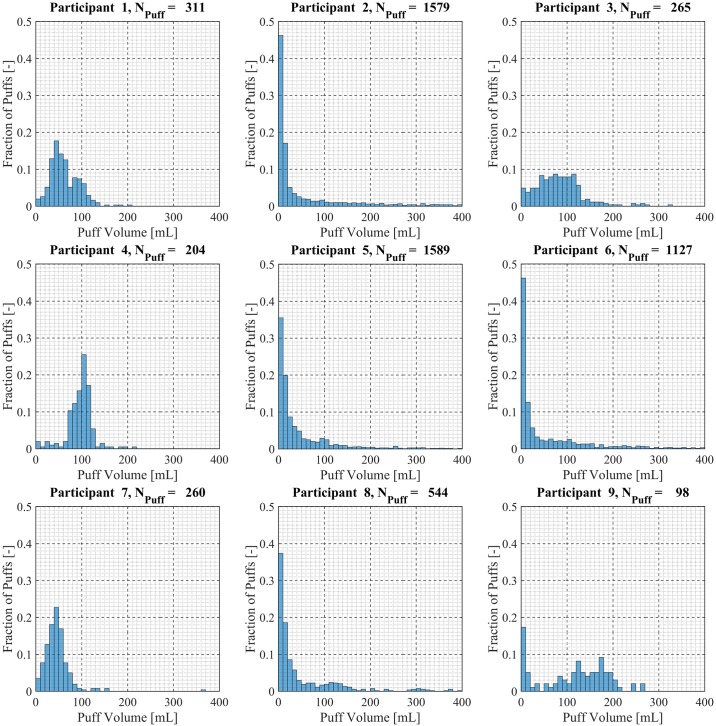
Frequency distributions of puff volume variation for participants 1–9. Each frequency distribution panel presents the variation of puff volume of a single participant across multiple puffing sessions conducted during *7 days* of ENDS use in the natural use enviroment.

**Fig 14:**
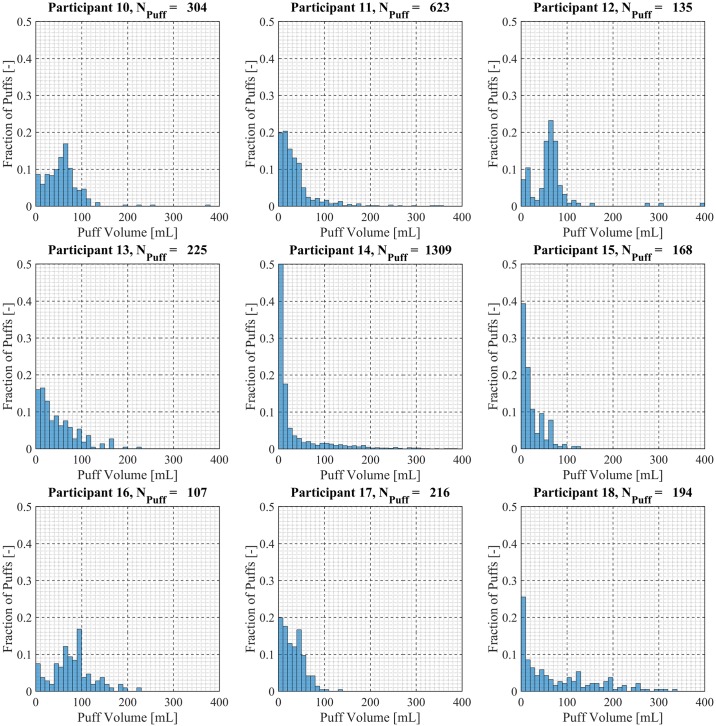
Frequency distributions of puff volume variation for participants 10–18. Each frequency distribution panel presents the variation of puff volume of a single participant across multiple puffing sessions conducted during *7 days* of ENDS use in the natural use enviroment.

**Fig 15:**
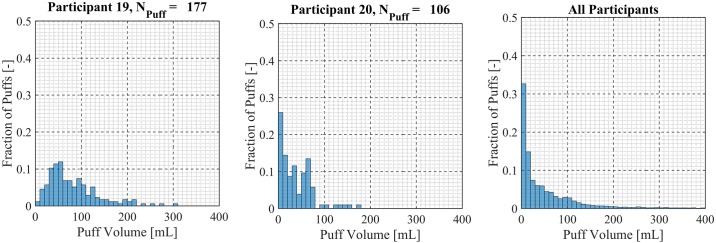
Frequency distributions of puff flow rate variation for participants 19–20. Each frequency distribution panel presents the variation of puff volume of a single participant across multiple puffing sessions conducted during *7 days* of ENDS use in the natural use enviroment. The right column panel presents puff volume variation for the entire cohort of 20 participants.

Participant-specific frequency distributions of puff duration, flow rate and volume were used to evaluate intra-participant variability exhibited by each of the *20* Participants over the course of the *7 day* observation period. 98.5% of puffs were observed to have durations less than *20 seconds*, puffs with durations longer than *20 seconds* were considered outliers and omitted from this analysis. The total number of puffs omitted was 153 (1.6%) of the 9694 puffs initially identified. Puff duration frequency distributions are shown in Fig 7 for participants 1 through 9, Fig 8 for participants 10 through 18 and Fig 9 for participants 19 and 20. Also shown in Fig 9 is the combined puff duration frequency distribution for all puffs taken during the 1-week session by all participants. Puff flow rate frequency distributions are show in Fig 10 for participants 1 through 9, Fig 11 for participants 10 through 18 and Fig 12 for participants 19 and 20. Also shown in Fig 12 is the combined puff flow rate frequency distribution for all puffs taken during the 1-week session by all participants. Puff volume frequency distributions are shown in Fig 13 for participants 1 through 9, Fig 14 for participants 10 through 18 and Fig 15 for participants 19 and 20. Also shown in Fig 15 is the combined puff volume frequency distribution for all puffs taken during the 1-week session by all participants.

The extent of intra-participant variability appears to be participant dependent, with some participants exhibiting a relatively wide spread in topography characteristics while other have relatively low variation. The degree of intra-participant variability for each topography characteristic is quantified by participant-specific standard deviations provided in Table 4. Participant-specific standard deviations range from *0*.*8* to *4*.*1 seconds* for puff duration, *6* to *23 ml/s* for puff flow rate and *24* to *164 ml* for puff volume.

**Table 4:** Mean topography characteristics for participants 1 through 20.

	Mean Puff Duration *(s)*			Mean Puff Flow rate *(mL/s)*			Mean Puff Volume *(mL)*		
Participant	Mean	SE Mean	Std. Dev.	Mean	SE Mean	Std. Dev.	Mean	SE Mean	Std. Dev.
1	2.0	4.2E-02	0.7	30	3.9E-01	7	63	1.8E+00	31
2	2.0	7.5E-02	3.0	24	3.0E-01	12	66	2.9E+00	114
3	2.4	7.4E-02	1.2	34	6.3E-01	10	84	3.2E+00	52
4	2.5	5.5E-02	0.8	40	8.4E-01	12	97	2.0E+00	29
5	2.0	7.5E-02	3.0	26	2.6E-01	10	61	2.9E+00	116
6	2.0	9.1E-02	3.1	27	3.7E-01	12	65	3.3E+00	110
7	1.3	3.8E-02	0.6	37	8.7E-01	14	47	1.9E+00	31
8	2.0	1.1E-01	2.5	22	4.0E-01	9	54	3.8E+00	89
9	2.5	1.4E-01	1.4	40	1.7E+00	17	114	7.4E+00	73
10	1.9	1.0E-01	1.8	32	6.6E-01	11	60	2.8E+00	49
11	1.4	6.9E-02	1.7	28	3.8E-01	9	42	2.9E+00	72
12	3.4	3.5E-01	4.1	27	6.0E-01	7	105	1.4E+01	164
13	2.0	9.3E-02	1.4	22	5.0E-01	8	48	2.8E+00	42
14	1.6	6.5E-02	2.4	19	2.1E-01	7	37	1.8E+00	67
15	1.1	6.0E-02	0.8	19	8.1E-01	11	24	1.9E+00	24
16	2.6	1.3E-01	1.3	30	8.7E-01	9	80	4.3E+00	45
17	1.3	5.5E-02	0.8	23	4.4E-01	6	33	1.6E+00	24
18	2.5	2.2E-01	3.1	33	1.5E+00	20	95	8.3E+00	115
19	1.4	7.6E-02	1.0	60	1.7E+00	23	88	6.8E+00	90
20	1.4	2.3E-01	2.4	35	1.2E+00	12	46	6.3E+00	65
Cohort Ave.	2.0	1.1E-01	1.8	30.4	7.3E-01	11.4	65.4	4.1E+00	70.1
Std. Dev.	0.6	7.6E-02	1.0	9.2	4.5E-01	4.3	24.8	3.0E+00	37.8
SE Mean	0.1	1.7E-02	0.2	2.0	1.0E-01	1.0	5.5	6.7E-01	8.5
Cohort Min.	1	3.8E-02	0.6	19	2.1E-01	6	24	1.6E+00	24
Cohort Max.	3	3.5E-01	4.1	60	1.7E+00	23	114	1.4E+01	164

Mean puff duration, mean puff flow rate and mean puff volume with standard error of the mean and standard deviation of each quantity for 20 participants across multiple puffing sessions conducted during *7 days* of ENDS use in the natural use enviroment. The cohort average, standard deviation, standard error of the mean, cohort minimum and cohort maximum are reported for each quantity.

### Inter-participant Variability

Inter-participant variability was evaluated by comparing the participant-specific mean topography characteristics across the 20 participant cohort. Participant-specific mean topography characteristics are listed in Table 4. Means were calculated for each participant based on the total puffs recorded and analyzed over the *7 day* observation period. All puffs over *20 sec* in duration were omitted for reasons mentioned previously. The observed standard error of the means (SE Mean) suggests the data observed for each participant over the *7 day* period was sufficient to characterize the mean values.

**Fig 16:**
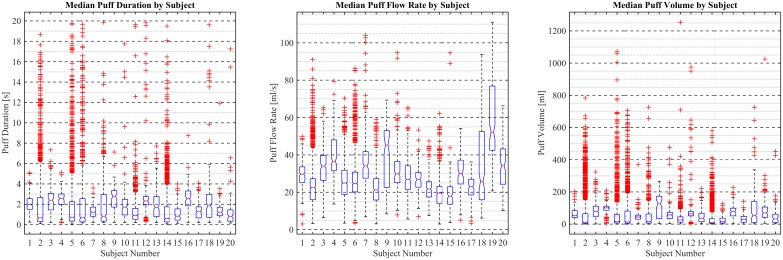
Boxplots of topography characteristic variation between participants 1 through 20. Each boxplot panel presents the median, 25^th^ percentile lower hinge, 75^th^ percentile upper hinge, 95% confidence interval on the median notch, and outliers beyond the upper and lower fence to illustrate the variation of puffing characteristics between 20 participants across multiple puffing sessions conducted during *7 days* of ENDS use in the natural use enviroment. The left panel presents puff duration, the middle panel presents puff flow rate, and the right panel presents puff volume. Individual standard deviations were used to calculate confidence intervals.

Boxplots are presented in Fig 16 to illustrate intra- and inter-participant variability in the median topography characteristics for Participants 1 through 20. Each box shows the intra-participant variability, while comparing the boxes to one another gives a sense of the inter-participant variability. The 95% confidence interval (CI) on the median value of each quantity is indicated by the notch in each box. Some users exhibited significant skew in puff duration characteristics (Participants 2, 5, 6, and 8), skew in puff flow rate (Participants 4, 6, 9, 18, and 19) and corresponding skew in the resultant puff volume (Participants 2, 5, 6, 8, 9, 13, 18, and 19). Median puff durations ranged from *0*.*5 sec* to *2*.*5 sec*, with a cohort mean of 2.0 (SE Mean 0.1). Median puff flow rates ranged from *17 mL/sec* to 52 *mL/sec*. *with a cohort mean of 30*.*4 (SE Mean 2*.*0)*. Median puff volumes ranged from 24 *mL* to *120 ml*, *with a cohort mean of 65*.*4 (SE Mean 5*.*5)*.

**Fig 17:**
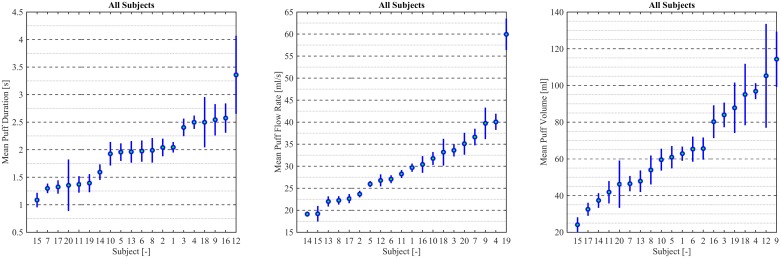
Interval plots of topography characteristic variation between participants 1 through 20. Mean puff duration sorted from smallest to largest with *95%* confidence interval around the mean for 20 participants across multiple puffing sessions conducted during *7 days* of ENDS use in the natural use enviroment. The left panel presents puff duration sorted from shortest to longest mean, the middle panel presents puff flow rate sorted from shortest to longest mean, and the right panel presents puff volume sorted from smallest to largest mean. Individual standard deviations were used to calculate confidence intervals.

Interval plots are presented in Fig 17 for *95%* CI in the mean to illustrate the inter-participant variability in mean topography characteristics. Mean puff durations ranged from *1*.*0 sec to 3*.*0 sec*, mean puff flow rates ranged from *24 mL to 114 mL* mean puff volumes ranged from *19 mL/sec* to *60 mL/sec*. A pair-wise 2-sided *t-*test comparison of topography characteristics between participants, as reflected by the interval plots, indicate significant variations in means across the cohort for puff duration, flow rate and volume. This complete set of *t*-test comparisons was conducted to explore potential unknown, or not predicted, patterns in participant vaping behavior. The left panel of Fig 17 suggests three possible groupings of mean puff duration, with a “short” mean puff duration of approximately *1*.*8 sec*, a “moderate” mean puff duration of approximately *2 sec*, and a “long” mean puff duration of approximately *2*.*5 sec*. The mean puff flow rate, illustrated in the middle panel of Fig 17, did not suggest similar grouping of behavior by the same participant cohorts. The resulting variation in mean puff volume, shown in the right panel of Fig 17 is inconclusive regarding participant groupings for mean puff exposure volume. Further investigation, with larger sample sizes of participants and knowledge of potential grouping parameters (such as gender, prior use history, ENDS device type, etc.) is necessary to fully characterize inter-participant topography variability.

### Frequency of Use and Cumulative Exposure from ENDS

The TAP was used to quantify cumulative exposure for each of the 20 participants. Table 5 presents the total number of vaping sessions, total number of puffs and total volume of e-liquid aerosol inhaled per participant as captured in this *7-day* study. An effective number of monitoring days was determined by reducing the observational period from *7 days* in proportion to the number of files included as input to the TAP (See Table 3) for each participant based on a linear regression. For example, Participant 1 was effectively monitored for *7 days* because 100% of Participant 1 files were included in the topography statistics, whereas Participant 2 was effectively monitored for 5.7 days because 82% of Participant 2 files were included in the topography statistics. The total number of vaping sessions, number of puffs and total puff volume observed for each participant were normalized by the number of effective days of monitoring data. Normalized daily exposure data can be compared to better understand the range of daily vaping sessions, daily puffs, and daily e-liquid aerosol exposure observed among the 20 participant cohort.

**Table 5:** Cumulative ENDS use and e-liquid aerosol exposure for participants 1 through 20.

Participant	*Effective* number of days monitored *(day)*	Cumulative exposure during observation period			Average exposure *per effective day*		
		Vaping sessions *(-)*	Total puffs *(-)*	Volume of e-Liquid aerosol *(mL)*	Sessions *(-/day)*	Number of puffs *(-/day)*	Volume of e-Liquid aerosol *(ml/day)*
1	7.0	30	311	19540	4	44	2791
2	5.7	88	1579	103602	15	275	18053
3	7.0	31	265	22248	4	38	3178
4	7.0	8	204	19765	1	29	2824
5	6.9	211	1589	96784	31	230	14038
6	5.8	63	1127	73588	11	196	12777
7	3.6	16	260	12095	4	72	3354
8	6.5	56	544	29352	9	83	4497
9	7.0	17	98	11198	2	14	1600
10	7.0	12	304	18109	2	43	2587
11	6.4	50	623	26058	8	98	4095
12	4.6	35	135	14208	8	29	3074
13	6.3	9	225	10757	1	36	1708
14	5.9	53	1309	48850	9	223	8313
15	7.0	18	168	4048	3	24	578
16	6.3	17	107	8585	3	17	1356
17	7.0	21	216	7026	3	31	1004
18	6.6	25	194	18441	4	29	2799
19	7.0	11	177	15546	2	25	2221
20	7.0	17	106	4897	2	15	700
Cohort Ave.	6.4	39	477	28235	6	78	4577
Std. Dev.	0.9	45	487	28660	7	81	4735
SE Mean	0.2	10	109	6409	1	18	1059
Cohort Min.	3.6	8	98	4048	1	14	578
Cohort Max.	7.0	211	1589	103602	31	275	18053

Cumulative electronic cigarette use and e-liquid aerosol exposure observed in the user’s natural environment for 20 participants across multiple puffing sessions conducted during *7 days* of ENDS use in the natural use enviroment. Also included is average effective daily exposure when accounting for fraction of collected data files analyzed with the topography analysis program.

The effective number of vaping sessions per day ranged from *1* to *31*, with a cohort-mean of *6 ± 1 sessions* (mean ± SE Mean). The effective total number of puffs ranged from *14 puffs/day* to *275 puffs/day*, with a cohort-mean of *78 ± 18 puffs/day* (mean ± SE Mean). The effective volume of e-liquid aerosol inhaled per day ranged from *0*.*6 liters* to over *18 liters*, with a cohort-mean of *4*.*6 ± 1*.*1 liters* (mean ± SE Mean).

As a first approximation, for those participants with *less than 7 days* of monitoring, the effective *per-day* participant averages may be extrapolated to estimate a weekly exposure. For those participants with *7 days* of effective monitoring, the data provided in Table 5 represent the actual weekly exposure.

The total number of vaping sessions presented in the third column of Table 5 is 788 sessions. Recall there were 1022 candidate data files recorded with the wPUM. The preliminary data analysis excluded 100 data files as described previously. The remaining 922 data files were analyzed with the TAP. TAP determined that 788 wPUM data files contained discernable puffs, while 134 files contained no observable puffs, indicative of the wPUM being turned on and then off again with no vaping activity. Only the 788 files containing observable puffs are reported as vaping sessions in the results presented.

## Discussion

Results from this study provide the first glimpse into how user behaviors vary over the course of a week in their natural environment. Significant intra-participant variability was observed for all three topography characteristics. Large standard deviations in participant-specific topography characteristics imply that there may be some affects related to time-of-day and day-of-the-week worth further investigation. Further evidence of such affects is seen by participant-specific frequency distributions exhibiting bi-modal distributions of topography data. Such detailed topography data, with standard deviations describing intra- and inter- participant variability inform human participant study design protocols for product safety testing, switching studies or initiation patterns of naïve users.

There may be a tendency to define topography protocols in terms of the “cohort-mean” values of puff duration, flow rate or puff volume. The possibility that ENDS aerosol production might be dependent upon both puffing topography itself (as a function of flow rate, for example) as well as the significant heterogeneity observed between individual participants suggests that it would be advantageous to utilize puffing protocols that encompass the range of user behaviors found in the natural environment. To that end, data from the current study lends itself to proposing such a protocol.

Table 6 illustrates that varying two factors (puff duration and flow rate) across six levels span the range of topography characteristics observed in the study sample, presented in Table 4 and reflected in Fig 16. The observed mean puff durations in Table 4 and Fig 16 range from a low of *0*.*6 (s)* to a high of *3*.*4 (s)* while the observed mean puff flow rates range from a low of *19 (mL/s)* to a high of *60 (mL/s)*, and the observed puff volumes range from a low of *24 (ml)* to a high of *114 (mL)*. Similarly, Table 6 provides representative puff durations, puff flow rates, and puff volumes which largely reflect the range of inter-participant variability observed in the study cohort. Table 6 suggests a range of values appropriate for a potential testing protocol for ENDS devices.

**Table 6:** Representative puff topography ranges observed in a sample of young adults using their preferred disposable ENDS.

	Representative Puff Flow Rates *(mL/s)*					
Representative Puff Duration *(s)*	20	25	30	35	40	45
**1.0**	20	25	30	35	40	45
**1.5**	30	38	45	53	60	68
**2.0**	40	50	60	70	80	90
**2.5**	50	63	75	88	100	113
**3.0**	60	75	90	105	120	135
**3.5**	70	88	105	123	140	158

Two factors, Mean Puff Duration *(s)* and Mean Puff Flow Rate *(mL/s)*, at six levels each yield Mean Puff Volumes *(mL)* which span the range of puffing topography characteristics observed in a cohort of twenty young adult ENDS users during a natural use environment, *7 day* observation period.

There is a tendency to estimate total exposure by total number of vaping sessions per day or total number of puffs per day. However, the ambiguous terminology for how users define a vaping session may lead to inaccurate self-reported exposures. Indeed, in the current study, it was observed that for some users a significant amount of time passed between “sets” of puffs, which one user might define as one session, whereas another might define as multiple sessions. The number of sessions computed in this study represents the current best estimate for number of sessions as reflected by number of files with actual puffing data. In reality, it is possible that continued analysis in the future will show that it is quite common for multiple puffing sessions to be present within a single data file, and this will give rise to the need to define what is meant by a session; where one session concludes and another begins. The current body of knowledge and results of the current study support the recommendation that the “number of vaping sessions” based on users’ self-reported data or based on monitoring data, should not be used as a measure of ENDS exposure.

Similarly, there is a tendency to estimate total e-liquid aerosol exposure by self-reported number of puffs taken per day. It is unknown whether ENDS users accurately recall the total number of puffs per day, but such a study is worth undertaking. However, even if the total number of puffs per day could be accurately recalled by the user, number of puffs alone does not quantify exposure in any meaningful way. Based on the results of the current study, it is recommended that total exposure be reported as the cumulative volume of aerosol inhaled over a defined period of time. This study demonstrated a methodical procedure for determining cumulative exposure of inhaled e-liquid aerosol based on precise measurement and rigorous analysis of topography characteristics.

While this study adds valuable data to the field of ENDS topography, there are several limitations unique to the study which are worth noting. (1) Only ‘cigalike’ devices were tested and therefore the topography presented should not be generalized to second or third generation devices. Indeed, as users move on to more sophisticated devices their topography should be re-assessed. (2) The study demographics were limited to college students between the ages of 18 and 21 and all but one participant was male. Therefore, the topography and exposure results may not represent lifetime smokers using ENDS in an attempt to quit smoking. Further study is needed to determine the impact of demographics on topography and use behavior. (3) Although participants agreed to use the wPUM for every puff taken over the 7-day monitoring period, there was no secondary measure to guarantee compliance with the protocol. In addition, the impact of the wPUM itself on topography and behavior is not known. The investigators acknowledge that this creates some uncertainty in the topography characteristics and the estimated cumulative weekly exposure reported herein. (4) Some study participants reported being dual users of ENDs and conventional tobacco products. Therefore, the estimate of e-liquid consumed over the 7-day period is only part of the nicotine exposure for these users. A future observational study is planned in which dual use of products will be monitored.

Several important questions emerged as result of this study which warrant further investigation. (1) The study did not quantify the amount of nicotine consumed by each participant, since such an estimate requires knowledge of the transfer efficiency of each ENDs device utilized in the study [30] which is not yet available for reporting. (2) The study did not investigate the impact of smoking history on topography. Analysis of smoking history is out of scope for this article and are being analyzed in a future paper. (3) The authors anticipate that time-of-day patterns may emerge, but found the topic to be too complex to address in the current article. Current research is under way to study effective means for presenting the massive quantities of data resulting from such studies in a concise and statistically relevant manner. (4) Further study is needed to define and detect the number of unique sessions for ENDS users. This problem is unique to ENDS topography, since conventional cigarette sessions are easily defined by smoking one complete cigarette. Grazing is an ENDS user phenomenon in which users puff all day long. Significant future work is necessary to define a statistically relevant distinction between the time between individual puffs within a session and the time between sessions.

## Conclusions

Results of this study indicate significant intra- and inter-participant variability with regard to puffing topography characteristics, suggesting that multi-day monitoring periods are advantageous for characterizing ENDS use behavior. Results imply such monitoring is enhanced when carried out in the natural environment and that monitoring in the laboratory environment has inherent limitations. Realistic topography protocols presented in this study are recommended for machine-driven aerosol emissions testing of new products to inform regulatory science. Cumulative volume of inhaled e-liquid aerosol, calculated by direct measurement of puff duration and flow rate are more meaningful measure of exposure than counting puffs or counting sessions. Methods presented in this study permit robust statistical characterization of ENDS topography and exposure to e-liquid aerosol. The results provided in this study should not be generalized to other ENDS designs, brands, or demographic populations. Other ENDS device types and various brands of each ENDS device type should be tested and compared to determine the effect of device design on puffing topography, frequency of use and cumulative exposure.

## Supporting Information

S1 File Time history data for Participant 1, Log00218. This data file contains the flow rate and cumulative volume vs. time for Participant 1, as shown graphically in Figs 3 and 5, respectively.
(CSV)

S2 File Time history data for Participant 2, Log00228. This data file contains the flow rate and cumulative volume vs. time for Participant 2, as shown graphically in Figs 3 and 5, respectively.
(CSV)

S3 File Time history data for Participant 3, Log00241. This data file contains the flow rate and cumulative volume vs. time for Participant 3, as shown graphically in Figs 3 and 5, respectively.
(CSV)

S4 File Time history data for Participant 4, Log00228. This data file contains the flow rate and cumulative volume vs. time for Participant 4, as shown graphically in Figs 3 and 5, respectively.
(CSV)

S5 File Time history data for Participant 5, Log00241. This data file contains the flow rate and cumulative volume vs. time for Participant 5, as shown graphically in Figs 3 and 5, respectively.
(CSV)

S6 File Time history data for Participant 6, Log00251. This data file contains the flow rate and cumulative volume vs. time for Participant 6, as shown graphically in Figs 3 and 5, respectively.
(CSV)

S7 File Time history data for Participant 7, Log00273. This data file contains the flow rate and cumulative volume vs. time for Participant 7, as shown graphically in Figs 3 and 5, respectively.
(CSV)

S8 File Time history data for Participant 8, Log00253. This data file contains the flow rate and cumulative volume vs. time for Participant 8, as shown graphically in Figs 3 and 5, respectively.
(CSV)

S9 File Time history data for Participant 9, Log00536. This data file contains the flow rate and cumulative volume vs. time for Participant 9, as shown graphically in Figs 3 and 5, respectively.
(CSV)

S10 File Time history data for Participant 10, Log00521. This data file contains the flow rate and cumulative volume vs. time for Participant 10, as shown graphically in Figs 3 and 5, respectively.
(CSV)

S11 File Time history data for Participant 11, Log00328. This data file contains the flow rate and cumulative volume vs. time for Participant 11, as shown graphically in Figs 4 and 6, respectively.
(CSV)

S12 File Time history data for Participant 12, Log00322. This data file contains the flow rate and cumulative volume vs. time for Participant 12, as shown graphically in Figs 4 and 6, respectively.
(CSV)

S13 File Time history data for Participant 13, Log00377. This data file contains the flow rate and cumulative volume vs. time for Participant 13, as shown graphically in Figs 4 and 6, respectively.
(CSV)

S14 File Time history data for Participant 14, Log00560. This data file contains the flow rate and cumulative volume vs. time for Participant 14, as shown graphically in Figs 4 and 6, respectively.
(CSV)

S15 File Time history data for Participant 15, Log00559. This data file contains the flow rate and cumulative volume vs. time for Participant 15, as shown graphically in Figs 4 and 6, respectively.
(CSV)

S16 File Time history data for Participant 16, Log00378. This data file contains the flow rate and cumulative volume vs. time for Participant 16, as shown graphically in Figs 4 and 6, respectively.
(CSV)

S17 File Time history data for Participant 17, Log00408. This data file contains the flow rate and cumulative volume vs. time for Participant 17, as shown graphically in Figs 4 and 6, respectively.
(CSV)

S18 File Time history data for Participant 18, Log00626. This data file contains the flow rate and cumulative volume vs. time for Participant 18, as shown graphically in Figs 4 and 6, respectively.
(CSV)

S19 File Time history data for Participant 19, Log00638. This data file contains the flow rate and cumulative volume vs. time for Participant 19, as shown graphically in Figs 4 and 6, respectively.
(CSV)

S20 File Time history data for Participant 20, Log00392. This data file contains the flow rate and cumulative volume vs. time for Participant 20, as shown graphically in Figs 4 and 6, respectively.
(CSV)
